# First Aid in Dentistry

**Published:** 1914-06-15

**Authors:** 


### BIBLIOGRAPHY.

FIRST AID IN DENTISTRY. This is a small book of 150 pages, written by Dr. E. P. R. Ryan of the U. S. Army, who writes evidently out of his experience, and is intended as a brief guide in emergencies requiring dental treatment. It is written with the purpose that physicians and nurses not trained in dental technique may handle dental cases more successfully because of the suggestions here made. The cause and effects of oral sepsis and dental lesions is briefly given, together with relief treatment of such character as may be applied with instruments and appliances, the general surgeon would be likely to have at his command. Pulpitis, alveolar abscess, necrosis, fractures of the jaws, extractions, etc., are briefly but sufficiently considered for the purpose intended. It will be a good book for all forms of pioneering enterprises, and will be especially useful to army surgeons. It would be well if hospital nurses could be acquainted with it. It is published by P. Blakiston's Sons & Co., Philadelphia.

